# [^18^F]FDG-PET/CT in Hodgkin Lymphoma: Current Usefulness and Perspectives

**DOI:** 10.3390/cancers10050145

**Published:** 2018-05-18

**Authors:** Salim Kanoun, Cedric Rossi, Olivier Casasnovas

**Affiliations:** 1 Nuclear Medicine Department, Institut Claudius Regaud, 31100 Toulouse, France; 2 Centre de Recherches en Cancérologie de Toulouse (CRCT), UMR1037 INSERM, Université Toulouse III: Paul-Sabatier, ERL5294 CNRS, Université de Toulouse, 31100 Toulouse, France; cedric.rossi@chu-dijon.fr; 3 Hématologie Clinique, CHU Dijon, 21000 Dijon, France; olivier.casasnovas@chu-dijon.fr; 4 INSERM UMR1231, Université de Bourgogne Franche Comte, 21000 Dijon, France

**Keywords:** Hodgkin Lymphoma, [^18^F]FDG, PET/CT, staging, treatment response, interim PET, total metabolic tumor volume

## Abstract

Functional imaging using 18-fluorodeoxyglycose ([^18^F]FDG) positron emission tomography combined with computed tomography (PET/CT) has become a major imaging modality in Hodgkin lymphoma. This imaging modality allows for a significant improvement in staging, increased sensitivity, which involves differentiating residual tumors from fibrosis during assessment, and highly impacts treatment decisions into new PET-driven strategies. This review presents the main scientific data concerning the current applications of [^18^F]FDG-PET/CT in Hodgkin lymphoma at baseline, interim, and the end of treatment evaluation along with the main PET-driven trials for therapeutic decisions. The emergence of total metabolic tumor volume as a new functional prognostic factor will also be discussed.

## 1. Introduction

Over the last several decades, the prognosis of Hodgkin lymphoma (HL) has constantly improved thanks to new therapy regimens and strategies. Initially an almost incurable disease in 1940, Hodgkin lymphoma has become a type of cancer with one of the best prognoses [1].

Today, the main therapeutic approaches are based on two chemotherapy regimens including ABVD (doxorubicin, bleomycin, vinblastine, and dacarbazine) and BEACOPP (bleomycin, etoposide, doxorubicin, cyclophosphamide, vincristine, procarbazine, and prednisone). The later provides a better progression for survival but also increases long-term toxicity, which affects fertility and increases secondary leukemia [2,3].

Different therapeutic strategies have been developed using these two chemotherapy regimens, with a clear need for biomarkers capable of identifying patients with poorer outcomes for treatment intensification and when to limit toxicity for patients with a better prognosis.

While therapeutic approaches are enhanced, new major developments also appeared in medical imaging for staging and treatment response evaluation.

The introduction of functional imaging with 18-fluorodeoxyglycose ([^18^F]FDG) positron emission tomography combined with computed tomography (PET/CT) was a major development in medical imaging over recent decades. 

This new imaging modality holds a major place in treatment decisions and has removed the usual weaknesses of conventional imaging as well as introduced personalized therapeutic decisions. 

In this review, we will present the main current and future applications of [^18^F]FDG-PET/CT for staging, response assessment, and its impact in therapeutic decisions for Hodgkin lymphoma. 

## 2. Staging

### 2.1. Ann-Arbor Staging

Proposed in 1971, the Ann-Arbor staging was initially based on physical examination and planar radiography such as chest radiography or lymphangiography, which are no longer performed [4]. Shortly after, these planar and invasive imagings were replaced by 3D-Computed tomography (CT), which allows for better tumor detection and a full-body staging in a single-step procedure [5]. However, conventional imaging even with CT was still affected by several limitations, especially for bone marrow involvement, which still had to be evaluated through bone marrow biopsy.

In early 2000, functional imaging using [^18^F]FDG-PET/CT showed the ability to more accurately detect the extent of Hodgkin lymphoma especially in cases of extra-lymphatic involvement. Several papers compared the accuracy of [^18^F]FDG-PET/CT and conventional staging such as Hutchings et al. that included prospectively 99 HL patients and showed an upstage in 19% and a downstage in 5% of patients [6]. The respective sensitivity of CT and [^18^F]FDG-PET/CT were 82.6% and 92.2% for nodal staging and 37% and 72.7% for extra-lymphatic staging, respectively. The specificity was excellent for both modalities in all cases (97% to 99%). These differences of sensitivity also led to treatment modification in 9% of patients.

As shown by Hutchings et al., the increased sensitivity of [^18^F]FDG-PET/CT is particularly improved for spleen involvement and bone marrow involvement (sensitivity was 83% and 70% versus 36.8% and 13.3% for CT, respectively). 

These data are also consistent with several other publications showing an increased sensitivity of [^18^F]FDG-PET/CT from 10% to 20% compared to conventional CT, which leads to an upstaging rate of 10% to 40% and a modified treatment decision in up to 20% of patients [7].

Last but not least, when radiotherapy (RT) is planned (usually for Ann-Arbor Stage I-II), [^18^F]FDG-PET/CT staging also significantly influence the involved field radiotherapy compared to CT-based targeting [8].

### 2.2. Bone Marrow Involvement

Because of the limitations of conventional CT, bone marrow involvement detection using [^18^F]FDG-PET/CT has been specifically evaluated to challenge the invasive bone marrow biopsy diagnosis. 

As shown in different publications, [^18^F]FDG-PET/CT demonstrates a clearly higher rate of bone marrow involvement than conventional imaging and even more than the bone marrow biopsy [9,10]. This led to gold standard definition issues since each focal [^18^F]FDG uptake could not be confirmed by guided biopsy for definitive diagnosis due to practical and ethical reasons.

However, as shown in the literature, the number of patients showing a positive bone marrow biopsy with a negative [^18^F]FDG-PET/CT was very limited and never introduced any change of therapy. Moreover, it has been demonstrated that [^18^F]FDG bone marrow positivity lead to a significantly poorer prognosis, which suggests an increased sensitivity in bone marrow evaluation when compared to a bone marrow biopsy [11].

Today, the bone marrow biopsy is no longer required for bone marrow staging in Hodgkin lymphoma. [^18^F]FDG-PET/CT allows a single step, non-invasive procedure for staging of nodal and extra-lymphatic disease with a strong prognosis value. This saves patients from the invasive procedure [12].

## 3. Response Assessment

As shown previously, functional imaging introduced a significant improvement in staging Hodgkin lymphoma but [^18^F]FDG-PET/CT allowed for more important improvements in response evaluation when compared to conventional imaging.

Two response assessment timings are now documented in the literature, which include End-of-Treatment PET/CT after the first line of chemotherapy (±radiotherapy) and Interim PET/CT during the first line of chemotherapy, usually performed after the first two cycles.

### 3.1. Interpretation Criteria

Several interpretation criteria have been proposed for [^18^F]FDG-PET/CT interpretation. The first consensus was reached in 2007 by the Imaging subcommittee of the International Harmonization Project in which [^18^F]FDG-PET/CT end of treatment interpretation was based on the size of residual mass and compared to the mediastinum background or surrounding background [13].

**Figure 1:**
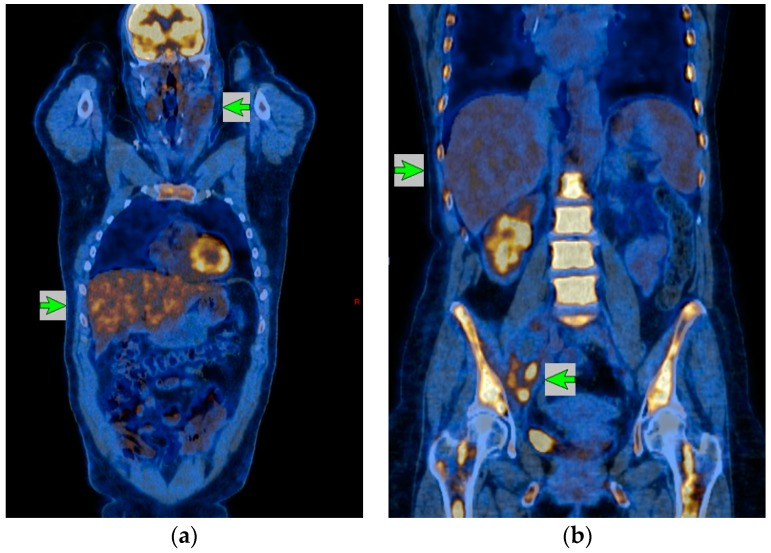
5-Points Scale (5PS)/Lugano classifications illustrations. Patient (**a**) has a residual mass with lower uptake than liver background and is, therefore, classified as 5PS = 3 and Complete Metabolic Response for Lugano classification. Patient (**b**) has a pelvic residual mass with an uptake clearly higher than the liver background and is, therefore, classified 5PS = 5 and Partial Response for Lugano classification. For each example, green arrows indicate the residual mass and the reference liver background.

While studies have shown that a fraction of PET positive patients still presented favorable outcomes, a more specific classification was proposed at the Deauville first international workshop defining the visual 5 Point Scale criteria (also called Deauville criteria) for both end of treatment and interim [^18^F]FDG-PET/CT treatment (see Figure 1 and Table 1) [14]. This scale is now widely documented in the literature.

A recent consensus reshaped this 5-point scale (5PS) in a newer classification called “Lugano Criteria” and relies on the same visual definition of uptake significance [12]. 

Five-point scale scores 1 to 3 are considered Complete Metabolic Response (CMR) while 5PS score 4 and 5 are considered either No-Metabolic-Response (NMR), Partial Response (PR), or Progressive Disease (PD) depending on the evolution compared to baseline evaluation (no change from baseline, reduced uptake compared to baseline, and increased uptake or new lesion, respectively).

This Lugano classification should now be used to standardize [^18^F]FDG-PET/CT reports since it has shown good prognosis discrimination and good inter-observer reproducibility [15].

### 3.2. End of Treatment

Before the [^18^F]FDG-PET/CT era, conventional CT evaluation suffered an inability to distinguish residual fibrotic masses from residual active tumors [16]. This limitation led to a high rate of unconfirmed response using conventional CT response evaluation, which has been removed with the [^18^F]FDG-PET/CT procedure.

Compared to conventional CT, [^18^F]FDG-PET/CT has shown a drastically higher prognosis value to identify patients with residual active disease who might benefit from salvage therapy [17,18]. 

As reported by Cerci et al., end of treatment evaluation using functional imaging had a sensitivity of 100%, specificity of 92%, Positive Predictive Value and Negative Predictive Value, respectively, of 92.3% and 100%, and it demonstrated a better cost-effectiveness than conventional imaging [19].

### 3.3. Interim PET

Early evaluation of treatment, usually performed after two cycles of chemotherapy (interim PET/CT) response, have shown a high prognosis value in several publications. This early evaluation leads to an evaluation of the chemo sensitivity of the tumor and, therefore, identifies patients with different outcomes at the first cycle of the first line of treatment.

For patients with an ABVD or BEACOPP regimen, [^18^F]FDG-PET/CT showing a residual tumor uptake after two cycles of chemotherapy (PET2) was predictive of a poorer outcome in progression free and overall survival [20,21,22]. 

This early PET/CT evaluation also outperforms the International Prognostic Index in patient’s prognosis identification. In published series, the [^18^F]FDG/PET/CT positivity rate appears consistent around 20% and is a strong predictor of a low Progresion Free Survival (PFS 27% versus 91% for PET2 negative patients) and also of a low Overall Survival (OS 62.5% versus 98.2%) [21]. 

Due to this ability of [^18^F]FDG-PET/CT to identify poorly responding patients, functional imaging has been evaluated in the PET-driven clinical trial strategy to optimize disease control or reduce treatment toxicity.

### 3.4. Immunotherapy Evaluation

Very recently, Program Death Inhibitor therapies have shown very unique response rates in relapsed or refractory Hodgkin lymphoma [23]. 

These new therapies are challenging imaging response evaluation since it introduces delayed response or pseudo-progression of the immune-related response. 

For these new therapies, new criteria called LYRIC (lymphoma response to immunomodulatory therapy criteria) have been defined and introduce delayed [^18^F]FDG-PET/CT evaluation to differentiate pseudo-progression from real disease progression [24].

The interpretation criteria of [^18^F]FDG-PET/CT for immunotherapy evaluation is not well validated yet. However, the first retrospective data available have shown accurate identification of disease progression using [^18^F]FDG-PET/CT and suggest new interpretation criteria based on global tumor burden evaluation [25].

## 4. PET-Driven Therapeutic Approaches

### 4.1. Early Stages

To avoid or at least to reduce long-term toxicities related to radiotherapy, [^18^F]FDG-PET-guided strategies in early stage HL have been evaluated recently in several large randomized trials. In the RAPID trial [26], 602 patients IA-IIA with HL and no mediastinal bulk received 3 cycles of ABVD, and 571 of them were evaluated by a PET/CT (PET3). Patients with a negative PET/CT (defined by Deauville score = 1 or 2) were a large portion of those patients (74, 6%, *n* = 426) and were randomly assigned to receive 30 Grays (Gy) involved field radiotherapy (IF-RT) or no further treatment. Among PET3 negative patients, 20 relapses occurred in the arm without radiotherapy versus only eight in the radiotherapy arm. Despite this relative excess of relapse into exclusively the chemotherapy arm, with a median follow-up of 60 months, the PFS at 3 years was 94.6% (95% CI 91.5–97.7%) in the radiation therapy arm versus 90.8% (95% CI 86.9–94.8%) in the arm without radiotherapy. The objective of non-inferiority between the two arms was reached (difference of 7% of PFS expected between the two arms to declare the non-inferiority).

The other randomized study [27] that attempted to answer the same question was conducted jointly by the European Organization for Research and Treatment of Cancer (EORTC), Lymphoma Study Association (LYSA), and Italian Lymphoma Foundation (FIL) (*n* = 1950 patients). This trial compared a standard ABVD arm followed by Involved Node Radiotherapy (INRT) 30 Gy to an experimental arm guided by PET/CT. Patients with negative PET2 received two or four additional cycles of ABVD depending on whether they were favorable or unfavorable according to EORTC/LYSA criteria without radiotherapy. With a median follow-up of 12 months at the time of the interim analysis, the trial was discontinued due to a higher number of relapses in the strategy without radiotherapy (16/258 [6.2%] vs. 7/251 [2.3%]). However, the final analysis with a median follow-up of 45 months had a favorable patient group where the difference of five-year PFS reached 12% between irradiated (99%) and non-irradiated patients (87%; Hazard-Ratio (HR) = 15.8). For patients in the unfavorable group, the difference in PFS was only 3% (92% vs. 89%, HR = 1.5) in favor of radiotherapy. Recently, a meta-analysis [28] showed that, with regard to these studies, there is a superiority of the combined chemo-radiotherapy strategy for tumor control (SSP): HR = 2.38 (95% CI) 1.38, 3.50).

Altogether, the chemo-radiotherapy combination gives better control of the disease (a loss in tumor control in early-stage favorable patients who are PET-negative after three or four ABVD with differences in 3-year and 5-year PFS of 3.8% and 11.9%, respectively) even though the large majority of patients can be cured with exclusive chemotherapy. In this very good prognostic population, the PET-guided RT strategy cannot be recommended. We lack long-term analysis to appropriately assess risks of late-stage toxicity of negative PET patients treated either by exclusive chemotherapy or chemotherapy combination radiotherapy. However, the critical question is to know if it is worth it to perform radiotherapy in all patients with the inherent risks of increased late toxicity while only a minority of them (about 5%) benefit from radiation therapy. Overall, the negativity of PET2 is probably not the appropriate criteria to make a decision on radiotherapy, and the difficulty remains to identify the small group of patients requiring radiation therapy. However, in the group with baseline unfavorable EORTC/LYSA criteria and negative PET2, 6-cycle ABVD provides similar PFS compared to four cycles of ABVD plus INRT and is an acceptable therapeutic option.

The H10 trial randomized positive PET2 patients [29] between two cycles of escalated BEACOPP (BEAesc) followed by INRT (30 Gy) or continuing ABVD followed by INRT (30 Gy). The results show that 14% and 25% of patients had a positive PET2 depending on whether they had a favorable or unfavorable disease at diagnosis, respectively. Patients of the BEAesc arm had a statistically better PFS than those who continued ABVD cycles (5-years PFS: 91% vs. 77%; HR = 0.42, 95% CI 0.23–0.74, *p* = 0.002). The rate of progressive disease was 18.8% in the ABVD arm compared to 7.7% in the BEAesc arm. There is also a trend to a better patient’s OS in the BEAesc arm (HR = 0.45, 95% CI 0.19–1.07, *p* = 0.062).

These results suggest that the treatment of patients with localized HL should be monitored by PET mainly to allow for intensifying the treatment of PET2 positive patients using BEAesc in order to improve the disease control in this subset of patients. This could be implemented in routine practice.

As mentioned previously, the Lugano/Deauville criteria are currently recommended for [^18^F]FDG-PET/CT interpretation while the HD10 trial was based on the older Juweid criteria, initially designed for end-of-treatment evaluation. The Deauville score was defined in 2009 to harmonize [^18^F]FDG-PET/CT interpretations in clinical trials (including interim evaluation) and demonstrated a good inter-reader reproducibility and a strong predictive value [14,15]. Therefore, the Deauville/Lugano classification appears applicable in routine practice for early stage Hodgkin lymphoma interim evaluation. Lastly, the RAPID trial excluded Deauville score 3 from PET negativity definition to select patients with the most favorable profile for treatment de-escalation, omitting radiotherapy, and this could appear in contradiction with the actual Lugano classification. However, the RAPID trial results do not support this de-escalation approach, which is why the [^18^F]FDG-PET/CT negativity defined by Deauville score 1–3 vs. 4–5 as proposed by Lugano classification still appears to be the best criteria for routine practice in Hodgkin lymphoma.

### 4.2. Advanced Stages

The International Prognostic Score (IPS), which involves the biological characteristics of tumor cells or of their microenvironment, determines that cytokine profiles circulating at diagnosis do not allow us to discriminate individually the patients who should be treated more intensively such as with BEAesc. Conversely, as in the localized stages, functional imaging allows us to study individually the chemo sensitivity of first-line treatment from two cycles [20] and to develop strategies adapted to risk. Two types of approaches have been investigated. 

The first so-called “escalation strategy” proposes strengthening the treatment for bad responders after two cycles of ABVD by introducing the BEAesc. An Italian phase II trial showed that the group of patients with positive PET after two cycles of ABVD receiving four cycles of BEAesc plus four cycles of baseline BEACOPP has a 62% 2-year PFS [30]. This group represents 14% of the 154 patients treated in this trial. This result was better when compared to historical series in which PET2+ patients pursuing ABVD had 45% 2-year PFS [31]. A Phase II trial of the SWOG S0816 tested the same strategy for PET2 positive patients [32] who received six cycles of BEAesc while negative PET2 patients pursued ABVD. With a median follow-up of 39.7 months, 18% of the 336 patients registered had a positive PET2 and a 64% 2-years PFS, which was significantly lower than the 82% 2-years PFS observed in negative PET2 patients. Very recently, these data have been confirmed in a phase III trial (GITIL/FIL HD 0607) including 782 patients with 19% positive PET2 associated with a lower 3-year PFS (60% vs. 87%) [33]. These results are in line with those of the RATHL Study [34] in which PFS at three years was 85.7% for PET2 negative patients treated with ABVD and 67.5% for those positive PET2 treated by BEAs or esc. In this latter study, the slightly better PFS observed in both PET2-positive and PET2-negative patients compared to the previous PET-driven study are probably related to more favorable patient populations with 41% Ann-Arbor stage II and only 37% IPS ≥ 3.

The second approach evaluated treatment de-escalation for good metabolic responders after two cycles of BEAesc (LYSA AHL2011 trial, GHSG HD18). As the negative predictive value of PET2 is significantly better than its positive predictive value, minimizing the risk to undertreat patients appears more attractive than intensifying treatment of PET2 positive patients.

The AHL2011 trial compared a standard arm in which patients receive six cycles of BEAesc to an experimental arm where patients received two cycles of BEAesc followed by cycles of ABVD for PET2 negative patients and four additional BEAesc cycles for PET2 positive patients. In the two arms, [^18^F]FDG-PET/CT after four cycles (PET4) was decisional. PET4 positivity was considered treatment failure. The goal of the study was to demonstrate the non-inferiority in terms of PFS of the experimental arm compared to the standard arm. The interim analysis showed that 87% of patients reached a negative PET2 (87% in the experimental and 88% in the standard arm) and after a median follow-up of 16.3 months [35], 2-year PFS was similar between both arms (91.6% versus 88.3%, *p* = 0.79). Prognostic analysis of the parameters influencing the PFS showed that only the results of PET2 and PET4 remain significant in multivariate analysis. Therefore, three prognostic groups were identified whose 2-year PFS was 95% (PET2−, PET4−), 78% (PET2+, PET4−), and 47% (PET4+). In terms of toxicity, the results showed a rate of serious adverse events significantly lower in the experimental arm (72% vs. 41%, *p* < 0.00001).

In the HD18 trial [36], the GHSG chose to randomly reduce the number of BEAesc cycles to 4 in PET2 negative patients and tested the addition of rituximab to BEAesc in the case of positive PET2. PET2 negativity included only Deauville score 1 and 2 while Deauville score 3 was considered as positive. Therefore, only 51% of patients had negative PET2 compared to 87% of the AHL2011 trial, which defined more specific positivity criteria (only Deauville 4 or 5 with a residual maximum Standardized Uptake Value (SUVmax) >140% of liver background to enhance inter-reader reproducibility). The Deauville score cutoff chosen in the HD18 trial was probably lacking specificity and, therefore, not relevant for identifying patients with a different outcome, since PET2 negative and positive patients had similar PFS. With a median follow-up of 33 months, the addition of rituximab did not improve the 3-year PFS in PET2 positive patients (93.2% vs. 92.7%) and in PET2 negative patients, four cycles of BEAesc provided a slightly better PFS and a significant OS improvement when compared to 6/8 cycles due to reduced toxic deaths. 

Altogether, should we start with ABVD or BEAesc in a guided PET strategy? Although it is difficult to make direct comparisons between studies, the results of the strategy beginning with BEAesc (AHL2011, HD18) appear to increase PFS by roughly 10% (73% to two years in the AHL trial, 90% at three years in the HD18 trial) of positive PET2 patients compared to the escalation strategy (GITIL/FIL HD 0607, SWOG S0816, and RATHL) with about 65% 3-years PFS. Similarly, for negative PET2 patients, the 2-year PFS rate is particularly high (>90%) and about 10% above the results observed in trials starting with ABVD.

## 5. Future Prognostic Index: Total Metabolic Tumor Volume

Recently, the Total Metabolic Tumor Volume (TMTV) has been introduced as a new functional parameter to predict patient prognosis at baseline. This parameter consists of tumor burden quantification on [^18^F]FDG-PET/CT baseline evaluation.

The tumor burden quantification strategy was historically proposed in the 1980–1990s and has major advantages from imaging enhancements. The tumor burden quantification was initially based on physical examination [37,38] and 2D radiography and lymphangiography. Then, it was based on 3D computed tomography [39], and now it can benefit from the increased [^18^F]FDG-PET/CT sensitivity with a direct access to active tumor mass quantification of both nodal and organ involvement. 

Several publications have shown a significantly worse prognosis in patients who have a high TMTV at baseline in Hodgkin lymphoma [40,41,42]. This new prognostic factor outperforms the IPS and the bulk definition (mass >10 cm). For early stage HL (Ann-Arbor I-II) TMTV differentiates two subgroups of the GHSG unfavorable group where one group with low TMTV has a prognostic close to the GHSG favorable group and the patients with high TMTV have a prognostic similar to advanced stages [42]. The TMTV prognostic value combined with interim PET allows identification of a new subset of patients with clearly different prognoses in PFS and OS survival [40,41]. Patients accumulating the two unfavorable factors with high TMTV at baseline and incomplete response after two cycles of chemotherapy presented a very poor prognosis (25% 5y-PFS for early stage patients [41]).

As availability and easiness of calculation appears crucial to implement this technique, free and open source software for PET/CT image processing with semi-automatic TMTV calculation are being proposed to develop the standardized software package and increase TMTV availability for researchers (http://petctviewer.org). However, this approach still needs better standardization, especially regarding the methodological segmentation rule and the TMTV cut-off definition [43]. 

This new functional parameter is calculated on the images from the usual [^18^F]FDG-PET/CT performed at baseline and does not require any additional procedure, acquisition, or cost. In the near feature, TMTV calculated on [^18^F]FDG-PET/CT may permit a routine use of tumor burden assessment, which has had a known value for decades but has not reached clinical applications yet.

## 6. Conclusions

Currently, [^18^F]FDG-PET/CT is the main imaging modality for staging and monitoring response to treatment in Hodgkin lymphoma. 

This functional imaging removed the usual limitations of computed tomography to allow more accurate prognosis identification. Furthermore, [^18^F]FDG-PET/CT added more accurate staging for organ involvement and more accurate response evaluation with better characterization of residual mass by differentiating fibrosis from a residual tumor. 

Early evaluation of treatment response has shown a high prognosis value, and several recent phase III randomized prospective trials validated the PET-driven therapeutic strategy, which has allowed a new area of personalized medicine and optimized disease control and toxicity for each patient. 

If [^18^F]FDG-PET/CT is now a mature modality included in most Hodgkin lymphoma guidelines, new PET/CT metrics are also in development to evaluate patients under new immunotherapy regimens and to bring an additional prognosis factor to predict patients’ outcomes. 

## Figures and Tables

**Table 1:** 5PS and Lugano criteria definition.

5PS	Definition	Lugano
1	No uptake	CMR
2	Uptake ≤ mediastinum	CMR
3	Uptake > mediastinum, but ≤ liver	CMR
4	Uptake moderately above liver	PR/NMR/PD
5	Markedly increased uptake above liver and/or any new lesion	PR/NMR/PD

CMR = Complete Metabolic Response; PR = Partial Response; NMR = No Metabolic Response; PD = Progressive Disease.
